# A Developmental Psychopathology Perspective on Adverse Childhood Experiences (ACEs): Introduction to the Special Issue

**DOI:** 10.1007/s10802-023-01100-w

**Published:** 2023-07-08

**Authors:** David J Hawes, Jennifer L Allen

**Affiliations:** 1 School of Psychology, University of Sydney, Camperdown NSW, 2006 Australia; 2 Department of Psychology, University of Bath, Bath, UK

**Keywords:** Adversity, Trauma, Child, Adolescent, Transdiagnostic, Caregiving

## Abstract

Adverse childhood experiences (ACEs), including child maltreatment and other adversities in the home context and beyond (e.g., witnessing domestic violence; parental mental illness; parental separation; living in a disadvantaged neighborhood) are prevalent in the population and often covary together. Research based on the construct of ACEs has transformed the field of adult mental health, yet child and adolescent mental health has often been overlooked in this work. This special issue of *Research on Child and Adolescent Psychopathology* focuses on the developmental science of ACEs and child psychopathology. The research presented here draws on the extensive evidence base that now exists regarding the co-occurrence of common childhood adversities, while informing the integration of theory and research on ACEs with that of developmental psychopathology at large. This Introduction provides an overview of ACEs and child mental health from a developmental psychopathology perspective, with an emphasis on key concepts and recent progress spanning the prenatal period through to adolescence and intergenerational pathways. Models of ACEs that emphasize the multi-dimensional nature of adversity and the importance of developmental timing to risk and protective pathways, have played a driving role in this progress. Methodological innovations in this work are highlighted, along with implications for prevention and intervention.

It is now well known that a broad range of early adversities can lead to deleterious outcomes later in life, and that the consequences of this adversity can be far reaching. Adverse childhood experiences (ACEs), including child maltreatment (e.g., physical abuse, neglect) and other adversities in the home context and beyond (e.g., witnessing domestic violence; parental mental illness; parental separation; living in a disadvantaged neighborhood) are prevalent in the population and often covary together. Such adversities are associated with increased risk for a broad range of physical and mental health problems, including not only trauma and stressor-related disorders, but the most common forms of psychopathology (Felitti et al., 1998; Hughes et al., 2017). It is important to recognize, however, that research based on the construct of ACEs has focused overwhelmingly on samples of adults (Struck et al., 2021), and has therefore often overlooked the mental health of children and adolescents.

The developmental science of ACEs and child psychopathology has now emerged as a distinct focus of the literature, as reflected in the articles published in the current special issue of *Research on Child and Adolescent Psychopathology*. This work draws on the extensive evidence base that now exists regarding the co-occurrence of common childhood adversities, while informing the integration of theory and research on ACEs with that of developmental psychopathology at large. Here we provide an overview of ACEs and child mental health from a developmental psychopathology perspective, with an emphasis on recent progress spanning the prenatal period through to adolescence and intergenerational pathways.

## Conceptualising ACEs Within Developmental Psychopathology

### Defining Adversity

In most research to date, ACEs have been operationalized using the adversities examined in the seminal ACE Study, and indexed using the ‘ACE score’ produced by summing the number of those experiences (Felitti et al., 1998). Approaches to the definition and conceptualisation of ACEs have nonetheless evolved considerably over time, and much has been written about the limitations of relying on the ACE score for this purpose (Lacey & Minnis, 2020). It is noteworthy that no formal definition of adversity was provided in the original ACE study, and there was no theoretical basis to the selection of the specific adversities included. Moreover, subsequent research has continued to suffer from a lack of consistent definitions. There is much to be gained by the collective adoption of a definition that addresses both developmental and ecological aspects of adversity, such as that proposed by McLaughlin (2016), which defines childhood adversity as experiences that are likely to require significant adaptation by a typical child and that represent a deviation from the expectable environment (i.e., the environmental inputs that the human brain requires to develop normally).

### The ACE Construct

The adversities specified in the original ACE Study relate largely to the micro- (individual) and meso-(family) system levels of influence articulated in ecological models of human development (e.g., Bronfenbrenner, 1986). These adversities span three broad categories of abuse (emotional, physical, sexual), neglect (emotional, physical), and household dysfunction (caregiver mental illness, substance use, incarceration, domestic violence, separation or divorce) (Felitti et al., 1998). Given that some experiences of adversities such as divorce and parental psychopathology will not meet the definition of adversity proposed, McLaughlin (2016) argued that they should only be indexed as ACEs when resulting in significant disruptions to caregiving or other significant adversity (e.g., consistent unavailability and unresponsiveness; emotional abuse).

Research based on an expanded approach to operationalising the ACEs construct has incorporated a greater emphasis on exo-level (extra-familial) adversities, including racial discrimination, placement in foster care, living in a disadvantaged neighborhood, witnessing violence, and bullying (e.g., Cronholm et al., 2015). It has been further argued that crisis migration (e.g., relocation due to fleeing large-scale emergencies such as armed conflicts, natural disasters, government repression) should also be recognized as an ACE, given its potential to disrupt development in many populations worldwide (Ertanir et al., this issue). Much remains to be learned about the relative contribution of these adversities to child mental health. Folk et al. (this issue), for example, found that while the original ACEs predicted behavioral outcomes among justice-impacted youth, this prediction was not significantly enhanced by expanded ACEs. This likely reflects the more proximal effects of micro- (individual) and meso- (family) systems on developmental pathways.

### Cumulative Risk and Dimensional Models

Implicit in research based on an overall ACE score is the assumption that discrete forms of adversity have additive effects on developmental outcomes, and that no single adversity is more important or influential than another. Although many studies have reported a roughly stepwise progression in risk based on the ACE score, findings have been highly heterogeneous (Hughes et al., 2017), and it has been estimated that about 30–40% of variance in outcomes is accounted for by additive synergistic interactions between certain pairs of individual ACEs (Briggs et al., 2021). To understand the contributions of ACEs to development and mental health it appears important to distinguish between different types of adversity, and various approaches to this can be found in the literature. Weems et al. (2021), for example, emphasized the distinction between ACEs that meet diagnostic criteria to be classified as traumatic stressors (e.g., physical abuse; sexual abuse; witnessing violence; natural disasters) versus those that typically do not (e.g., emotional abuse; caregiver mental illness; caregiver incarceration). This recognizes the importance of how adversities are subjectively experienced, which in the context of traumatic stressors includes perceptions of extreme threat (e.g., potential death, serious injury, sexual violence; American Psychiatric Association, 2013).

Researchers informed by evolutionary life-history theory have distinguished between dimensions of adversity related to unpredictability and harshness (Ellis et al., 2022). Unpredictability in early caregiver-child interactions (e.g., lack of contingent responsivity to infant cues), shapes children’s expectations of the environment and undermines not only the formation of secure attachment, but broader cognitive and socioemotional development (Ugarte & Hastings, 2022). Unpredictability in the child’s broader ecology, such as variability in housing, parental employment, and parental involvement in care, may further affect development by disrupting caregiving behavior. Conversely, harshness is an overarching concept encompassing experiences spanning threat and deprivation. McLaughlin et al. (2014) further conceptualized threat and deprivation as separate dimensions. Threat experiences are those associated with actual harm or threat of harm to survival, including direct victimization (e.g., physical abuse), as well as victimisation witnessed by the child (e.g., interparental violence). Deprivation involves a lack of social and cognitive inputs from the environment that reduces opportunities for learning (e.g., neglect). As discussed further in the following sections, these models recognize that experiences of adversity are often complex and co-occurring, while attempting to distill these experiences into core underlying dimensions that cut across multiple forms of adversity and shape learning and patterns of brain development in distinct ways.

Although ACEs increase risk for many childhood disorders, these disorders often also develop in the absence of adversity. This reflects the principle of equifinality in developmental psychopathology, whereby a single outcome may originate from different risk factors (Cicchetti & Rogosch, 1996). For example, adversity may feature heavily in the risk pathway to oppositional defiant disorder in one child, whereas this same disorder may arise from largely separate risk factors in another (Hawes et al., in press). Multifinality, conversely, refers to the multiple outcomes that may result from the same risk factor, as reflected in the broad range of disordered and healthy outcomes that can follow from ACEs. It has been argued that dimensional models of ACEs are particularly key to identifying the risk mechanisms that may account for such multifinality, and likewise, informing targeted intervention and prevention strategies (McLaughlin, 2016; Weems et al., 2021).

These conceptualisations have begun to inform novel studies of ACEs and child mental health involving a range of innovative methodological approaches. This includes research by Sisitsky et al. (this issue), in which person-centred analysis was used to identify latent profiles based on levels of threat and deprivation experienced by children at age 3 years, and found that later externalising and internalising symptoms (age 9) were best captured by a model that included four distinct types of exposure (home threat, community threat, lack of stimulation, and neglect). Similarly, in a longitudinal study of children (mean age 9 years) followed into early adulthood, latent profiles based on type of ACEs (Low Exposure, Familial Dysfunction, Emotional Maltreatment, Pervasive Exposure), were differentially associated with externalising and internalising outcomes (Nguyen et al., this issue). Research further suggests that the distinction between such dimensions may help explain intergenerational risk pathways. For example, Lyons-Ruth et al. (this issue) found that threat versus deprivation dimensions of mothers’ own ACEs were differentially associated with patterns of brain development in their infants. Specifically, a maternal history of childhood neglect, but not abuse, was associated with lower infant grey matter volume, whereas abuse, but not neglect, was associated with smaller infant amygdala volume later in development.

## Trajectories of Typical and Atypical Development

### Risk Mechanisms

The mechanisms by which ACEs lead to child psychopathology may at times be overlapping and embedded within one another, due to the systemic nature of the family environment and its central importance to development. Within this context, parent-child relationship processes (e.g., parent-infant synchrony; attachment security; emotion socialisation; coercive cycles; parental monitoring) are particularly proximal influences on the child, and it is largely through such processes that factors in the broader ecology of the child are understood to confer risk (Bronfenbrenner, 1986; Hawes et al., 2021a). It is therefore noteworthy that ACEs encompass not only extreme forms of adverse parenting (e.g., physical abuse; neglect), but also a range of known determinants of parenting and moderators of parenting influences (e.g., parent mental health; interparental conflict; socioeconomic disadvantage) (Taraban & Shaw, 2018). Via such pathways, ACEs have the potential to interact and transact with the neurobiology of the developing child, altering corticolimbic circuitry and largescale brain networks underlying cognitive and affective mechanisms for psychopathology. These include the frontoparietal network involved in cognitive control, the frontostriatal network involved in reward processing, and the salience network involved in detection of behaviorally relevant stimuli. This circuity is implicated in a number of key transdiagnostic mechanisms, including emotion regulation, social information processing, and associative learning (McLaughlin et al., 2020).

Evidence further suggests that mechanisms vary according to type of ACEs, as emphasized in the threat-deprivation model (McLaughlin et al., 2014). Notably, ACEs involving threat have been associated primarily with disruptions to aversive learning and emotion processing, which appear to be accounted for in part by alterations in corticolimbic circuitry, including increased amygdala activation to threat and reduced amygdala, mPFC, and hippocampal volumes (Hein & Monk, 2017; Weissman et al., 2020). Conversely, ACEs involving deprivation have been more consistently associated with impairments in executive functioning and language (Johnson et al., 2021; Sheridan et al., 2017), which appear to be accounted for in part by altered function in frontoparietal regions and reduced gray matter volumes (Hanson et al., 2013).

Evolutionary–developmental accounts posit that early adversity directs or regulates biobehavioral systems toward patterns of functioning to meet the environmental demands that individuals are likely to encounter (Ellis et al., 2022). Over both evolutionary and developmental timescales, life history strategies are thought to coordinate physiology and behavior in ways that historically enhanced expected fitness within the constraints of a given environment, even if they no longer do in the contemporary environment. ACEs, are thought to influence learning and changes in brain circuits through experience-driven plasticity mechanisms that facilitate adaptation to the environment in which a child is developing, while inadvertently contributing to heightened risk for psychopathology as children mature. For example, threat-based adversities appear to alter the development of neural networks underlying salience detection and aversive learning in ways that facilitate rapid identification of potential danger and mobilize defensive responses that promote safety (McLaughlin et al., 2014). Although adaptive when faced with immediate danger, attentional biases of this kind carry long-term costs including vulnerability to internalizing and externalizing disorders (e.g., (Crick & Dodge, 1994; Willner et al., 2020).

In addition to risk mechanisms related to learning and neuroplasticity, evidence furthers points to potential pathways involving epigenetic, endocrine, microbiome, and immune processes, among others (Gee, 2021). This includes psychoneuroimmunology models of toxic stress (see Kautz, 2021 for a review). For example, a prospective study of inflammatory biomarkers in a diverse sample of adolescents by Kautz et al. (this issue) found support for an early-life stress sensitisation prediction, whereby early adversity appears to affect biological stress reactivity, while also priming the immune system to exaggerate responses to subsequent stressors. This is important, given the potential for elevated low-grade inflammation to shape neural architecture, cognitive functioning, and the progression of psychopathology (Kautz, 2021).

### Developmental Timing and Sensitive Periods

From a developmental psychopathology perspective, mental health problems reflect deviations from a healthy developmental trajectory over time (Sroufe, 1997). Accordingly, the transformations that occur as part of normative development are key to understanding the mechanisms that shape problem trajectories, and these mechanisms can vary markedly depending on stage of development. It follows that to understand how ACEs influence child mental health, the timing of such experiences must be considered. Emerging measurement tools that incorporate such timing have been used to show that ACEs within specific age-based periods of childhood explain unique variance in symptoms of psychopathology in clinic-referred and community samples of children, independent of the overall chronicity of those ACEs (e.g., Hawes et al., 2021b). Further, the way in which ACEs cluster together may vary across developmental periods. For example, a network analysis study identified two similar clusters comprising direct abuse and adverse family factors in childhood and adolescence networks, along with a third cluster (educational and social adversities) in adolescence only (Pollmann et al., 2022).

The neural circuitry sensitive to environmental influences undergoes dynamic changes from the prenatal period through to young adulthood, and neuroplasticity is understood to vary across development (Lenroot & Giedd, 2006). Accordingly, evidence regarding the developmental timing of adversity is especially relevant to identifying sensitive periods of emotional, social, cognitive, and neurobiological development when the influences of ACEs on subsequent mental health may be most pronounced. While adversity early in life has particularly strong effects on neurobiological development relative to that in adulthood, evidence points to complex interactions between developmental timing, type of adversity exposure, sex, and regional specificity in the brain (Gee, 2021). As such, some risk may be greatest when adversity occurs during specific windows of heightened plasticity later in development (Reh et al., 2020). For example, one study found physical maltreatment at 3–6 years to be associated with blunted amygdala reactivity, whereas peer emotional abuse during adolescence (ages 13 and 15) was associated with an increased amygdala response (Zhu et al., 2019).

Converging evidence from animal and human research points to time windows for sensitive periods regarding development of the hippocampus (before age 13), amygdala (most pronounced volume changes during childhood), PFC (before age 2), and HPA axis (before age 2) (Malave et al., 2022). While animal studies allow researchers to experimentally manipulate the timing of exposure to adversity, tests of these periods in humans are challenging. Naturalistic designs involving children exposed to extreme deprivation, such as institutional rearing, have proven highly informative in this regard. Studies of institutionally reared children subsequently placed into stable family care suggest that at around 2 years of age, quality of caregiving has unique effects on autonomic nervous system and hypothalamic-pituitary-adrenal (HPA) reactivity (McLaughlin et al., 2015). Importantly, research also found that the majority of these children formed secure attachments when placed into stable family care prior to age 2, but that most placed after did not (Smyke et al., 2010). This age appears to represent a sensitive period for the developing attachment system, and one in which the caregiving environment may be particularly likely to alter the development of stress response systems and shape aspects of emotional development downstream.

Brain development is understood to be particularly malleable during prenatal and early postnatal life, when complex neural circuits underlying cognition and behavior are formed and refined through an interplay of inhibitory and excitatory neural input, synaptogenesis, synaptic pruning, myelination, and neurogenesis (Malave et al., 2022). Maternal ACEs and negative life events during the prenatal period may disrupt these processes and influence mental health across infancy and childhood. For example, in a prospective study of mother-infant dyads followed across pregnancy and infancy, Barclay (this issue) found that maternal early life adversity predicted increased depressive symptoms during pregnancy, which in turn predicted dampened infant cortisol reactivity. Another prospective study suggested that adversity during pregnancy (e.g., negative life events, contextual, parental or interpersonal stressors), has negative effects on children’s sleep that persist across childhood, and are exacerbated by genetic liability for insomnia (Kocevska et al., this issue). Such findings highlight the role of ACEs in the intergenerational transmission of psychopathology and the importance of considering the prenatal environment in prevention strategies.

Studies of samples exposed to adversity during later periods of development have begun to contribute valuable insights into such risk mechanisms, yet are relatively novel. Brieant et al. (this issue), report that their study of cognitive control mechanisms may be the first to isolate the effects of adversity experienced during adolescence on the adolescent brain. While a composite of adversities during adolescence was associated with impaired performance on a cognitive control task, it was not associated with atypical neural activation during the task. This was unexpected, as adolescents with histories of adversity have exhibited atypical neural activation in previous research (e.g., Fava et al., 2019). Given that previous research has indexed exposure to adversity across development rather than during adolescence specifically, it is possible that sensitive periods for such effects earlier in childhood may account for these discrepant findings. This will be important to test in further studies that account for the developmental timing of adversity.

### Acceleration of Development

There is now compelling evidence that early adversity affects the timing or pace of neurobiological development itself, and accelerates biological aging across multiple domains (Belsky, 2019). Early pubertal timing is one widely studied example, which from an evolutionary perspective may be adaptive for reproduction in harsh environments. Early adversity may also lead to precocious maturation of corticolimbic circuitry and behaviors governed by this circuitry, thereby promoting the independent regulation of stress and emotion in the absence of nurturing, stable environment, as reflected in the stress acceleration hypothesis (Callaghan & Tottenham, 2016). Such effects may also vary according to characteristics of adversity. For example, a study of female adolescents found that abuse was associated with delayed structural maturation in emotion-related neural circuitry, whereas neglect was associated with a more distributed pattern of accelerated structural maturation (Keding et al., 2021). Additionally, recent meta-analytic research found that while threat-based experiences were associated with accelerated development in terms of pubertal timing and cellular aging, deprivation-based experienced were not (Colich et al., 2020).

Using DNA methylation markers, researchers have begun to examine epigenetic age acceleration (EAA) as it relates to adversity and child mental health. EAA has been proposed as a biomarker of physiological deterioration due to the chronic activation of stress response systems, and has been associated with prior exposure to violence (e.g., Sumner et al., 2019). Among mothers, EAA represents a potential mechanism of intergenerational risk, as investigated by McKenna et al. (this issue). In this study, maternal EAA (indexed during pregnancy) was found to predict emotional reactivity among offspring (at 3-year follow-up), in an African American sample. Interestingly, however, mothers’ EAA was not associated with their self-reported experiences of childhood adversity. While further research is needed to understand the reasons for this, the effects of adversity on EAA may be reliant on a range of factors, including developmental timing, which has previously been found to explain more variability in DNA methylation than the accumulation or recency of exposure (Dunn et al., 2019).

### Protective Influences and Implications for Intervention

Compared to what is known about the effects of ACEs on risk for child psychopathology, the factors that may protect against this risk, including gene ✕ environment interactions, are less clear (Hidalgo et al., 2022; McLaughlin, 2016). Meta-analytic research suggests that factors including self-regulation, family support, school support, and peer support, confer protective (additive and/or buffering) influences on child outcomes following exposure to forms of violence including maltreatment, intimate partner violence, and community violence (Yule et al., 2019). Findings nonetheless highlight the complexity of protective pathways, particularly among diverse populations of children and young people (Masten et al., 2021). For example, racial and ethnic minority youth are known to experience higher rates of adversity (e.g., witnessing and experiencing violence) than non-minority youth, yet numerous studies have found them to report fewer post-traumatic stress symptoms than non-minority-youth (see Herd et al., this issue). Accounts of such findings have emphasized the potential for ethnic and cultural socialisation to buffer youth against racial stress and trauma, yielding greater self-efficacy and coping skills (e.g., Racial Encounter Coping Appraisal and Socialization Theory; Anderson and Stevenson, 2019). Other research has found that while positive childhood experiences (PCEs; e.g., positive family, school, and community connectedness) moderate the impact of ACEs on youth psychopathology (e.g., Qu et al., 2022), such pathways may involve significant interplay among various types of adversity. For example, Thomas et al. (this issue) found PCEs to be negatively related to diagnosable problems in youth exposed to parental incarceration, yet PCEs were more protective for children without an incarcerated parent.

Research into neurobiological aspects of protective pathways has found that positive parenting buffers the effects of social disadvantage on HPA axis function and corticolimbic networks involved in executive control and emotion regulation (e.g., Whittle et al., 2017). In additional to correlational findings, RCTs of parenting interventions with infants, young children, and adolescents exposed to various adversities have provided growing causal evidence of neurobiological gains (e.g., Brody et al., 2017; Valadez et al., 2020). These gains are therefore not restricted to the earliest periods of development, and emerging theory suggests that adolescence may confer unique opportunities for recalibration of the HPA axis following early adversity, due to increased plasticity during puberty (DePasquale et al., 2021). Positive parenting practices are among the major targets of many evidence-based parenting interventions for child mental health disorders (Hawes & Allen, 2016), and the use of such interventions to promote resilience has received growing attention.

Interventions that promote positive parenting skills appear to be effective in preventing and reducing child maltreatment risks (Gardner et al., 2023), yet individual differences in response remain poorly understood and may potentially reflect differential susceptibility to the environment (van IJzendoorn et al., 2020). It is noteworthy that parents and practitioners in recent years have questioned whether limit-setting strategies such as ‘time-out’, commonly included in such interventions, risk retraumatizing children with previous adversity (Dadds & Tully, 2019). In a trial designed to test this in children referred for conduct problems, those with high exposure to ACEs exhibited equivalent, if not greater, improvement in emotional and behavioral adjustment from such an intervention compared to low exposure children (Roach et al., 2022). Evidence-based parenting interventions with time-out therefore appear well suited to trauma-informed practice. Additionally, some research suggests that school-based interventions targeting early social-emotional and language skills in the preschool period may buffer children from the negative impact of early ACEs on later adolescent adjustment (e.g., Sanders et al., 2020). However, effective and scalable interventions to prevent the emergence of psychopathology in children exposed to broader ACEs are subject to ongoing development (Rith-Najarian et al., 2021).

## Conclusions

Advances in the developmental psychopathology of ACEs have moved rapidly in recent years, and owe much to decades of programmatic multi-disciplinary work in related fields, particularly child maltreatment (Cicchetti, 2016). Key progress has been driven by models of ACEs that emphasize the multi-dimensional nature of adversity and the importance of developmental timing to risk and protective pathways. These pathways, however, remain poorly understood, particularly in culturally diverse populations. There is compelling evidence that positive parenting buffers children against the cascading risk processes initiated by ACEs, and parenting interventions may therefore be particularly central to the promotion of resilience. Ongoing innovation in research methods and designs, including the integration of neurobiological indices within intervention trials, will be needed to build on this progress.
